# Apoptosis-associated uncoupling of bone formation and resorption in osteomyelitis

**DOI:** 10.3389/fcimb.2013.00101

**Published:** 2013-12-19

**Authors:** Ian Marriott

**Affiliations:** Department of Biology, University of North Carolina at CharlotteCharlotte, NC, USA

**Keywords:** osteomyelitis, apoptosis, osteoblasts, osteoclasts, inflammation, osteoimmunology, bacterial infection

## Abstract

The mechanisms underlying the destruction of bone tissue in osteomyelitis are only now being elucidated. While some of the tissue damage associated with osteomyelitis likely results from the direct actions of bacteria and infiltrating leukocytes, perhaps exacerbated by bacterial manipulation of leukocyte survival pathways, infection-induced bone loss predominantly results from an uncoupling of the activities of osteoblasts and osteoclasts. Bacteria or their products can directly increase osteoclast formation and activity, and the inflammatory milieu at sites of infection can further promote bone resorption. In addition, osteoclast activity is critically regulated by osteoblasts that can respond to bacterial pathogens and foster both inflammation and osteoclastogenesis. Importantly, bone loss during osteomyelitis is also brought about by a decline in new bone deposition due to decreased bone matrix synthesis and by increased rates of osteoblast apoptosis. Extracellular bacterial components may be sufficient to reduce osteoblast viability, but the causative agents of osteomyelitis are also capable of inducing continuous apoptosis of these cells by activating intrinsic and extrinsic cell death pathways to further uncouple bone formation and resorption. Interestingly, bacterial internalization appears to be required for maximal osteoblast apoptosis, and cytosolic inflammasome activation may act in concert with autocrine/paracrine death receptor-ligand signaling to induce cell death. The manipulation of apoptotic pathways in infected bone cells could be an attractive new means to limit inflammatory damage in osteomyelitis. However, the mechanism that is the most important in bacterium-induced bone loss has not yet been identified. Furthermore, it remains to be determined whether the host would be best served by preventing osteoblast cell death or by promoting apoptosis in infected cells.

## Introduction

Osteomyelitis is a severe infection of bone tissue that is associated with significant morbidity and typically leads to bone resorption, dysfunction, and progressive inflammatory destruction (Sax and Lew, 1999). Such infections are characterized in rodent models by the rapid production of inflammatory mediators, followed by infiltration of leukocytes at 3–7 days after infection, and subsequent bone resorption and adjacent areas of new bone deposition at 14–28 days (Yoshii et al., 2002). *Staphylococcus aureus* and *Salmonella* spp. are the most common causative agents of osteomyelitis. *S. aureus* accounts for approximately 80% of all osteomyelitis cases (Lew and Waldvogel, 2004; Labbé et al., 2010) while *Salmonella* species represent one of the most serious pathogens of bone in sickle cell patients and immunosuppressed patients (Anand and Glatt, 1994; Workman et al., 1996; Koehler et al., 1998; Overturf, 1999). *S. aureus* has a propensity to colonize broken skin and so a history of trauma or skin infection is a significant risk factor for bone and joint infections caused by this organism (Barton et al., 1987; Dubey et al., 1988). The majority of bone infections in children are caused by hematogenous spread of bacteria from distant infection foci through the bloodstream, while most cases in adults result from external sources such as post-traumatic wounds and post-operative infections (Mousa, 2003; De Boeck, 2005). Indeed, implant-related infection is such a feared complication in orthopedic surgery that perioperative administration of antibiotics is routinely used to reduce this risk (Davis, 2005). However, despite prophylaxis and improvements in the diagnosis of osteomyelitis, the incidence and severity of these bone infections appear to be increasing (Jensen et al., 1997; Arnold et al., 2006).

While osteomyelitis is associated with progressive inflammatory tissue destruction, such infections also result in marked bone resorption at sites of infection and proximal abnormal bone formation. The continual process of bone remodeling requires the coordinated regulation of the genesis and activity of osteoblasts and osteoclast lineages. Osteoclasts drive the resorption of bone by acidification and release of lysosomal enzymes (Teitelbaum et al., 1997). In contrast, osteoblasts produce components of bone, principally type I collagen, and catalyze the calcification process. As such, any interference with these integrated cell types can result in abnormal bone remodeling. Bacteria such as *S. aureus* and their products can be potent stimulators of resorptive bone loss (Nair et al., 1995, 1996). While bacteria can directly damage bone by producing acids and proteases, they can also stimulate osteoclastogenesis. For example, the site of infection in animal models of *S. aureus* osteomyelitis contains high numbers of macrophages and osteoclasts (Wiggers et al., 2011), and *S. aureus* surface-associated proteins can stimulate osteoclast formation and activity (Meghji et al., 1998; Lau et al., 2006). Similarly, systemically administered lipopolysaccharide (LPS) or local application of LPS derived from *Aggregatibacter actinomycetemcomitans* can reduce bone volume (Ochi et al., 2010; Madeira et al., 2013) and macrophages and osteoclast-like cells respond to this Gram-negative bacterial product by releasing cytokines and nitric oxide (NO) (Wiggers et al., 2011). However, it is unclear whether such effects are due to a direct action on osteoclasts and/or their progenitors, or are secondary to the production of other mediators, such as inflammatory cytokines, which modulate osteoclast formation and activity (Meghji et al., 1998).

Importantly, it is becoming increasingly apparent that, in addition to increased osteolysis and direct inflammatory degradation of bone matrix, tissue damage during osteomyelitis progression is associated with significant bone cell death. Again, cultured macrophages and osteoclasts can respond to LPS and *S. aureus* by releasing factors such as NO that can promote cell death (Wiggers et al., 2011). Importantly, osteonecrosis of the jaw is characterized by high-grade inflammation and large lesions coupled with osteocyte apoptosis (Lesclous et al., 2009). The death of this cell type is especially important given that this bone matrix embedded cell type regulates the bone remodeling activities of bone surface osteoclasts and osteoblasts via the lacuna-canaliculi network (as discussed in Matsuo, 2009). Furthermore, apoptotic cells accompany trabecular osteolysis in porcine models of osteomyelitis, in a similar manner to that typically observed in osteomyelitic lesions in following hematological *S. aureus* spread (Jensen et al., 2010). In the present review, we provide an overview of the mechanisms that can directly or indirectly induce bone cell death following infection and we discuss the possible significance of modulated programmed cell death in the progression of osteomyelitis.

## Role of resident bone cells in inflammatory bone loss associated with osteomyelitis

Current treatment for osteomyelitis involves expensive and prolonged (even lifelong) parenteral antibiotic treatment, surgically debridement of necrotic bone, and often amputation. While these strategies are effective in reducing or eliminating the ability to isolate culturable bacteria from affected tissue, osteomyelitis is often refractory with a recent retrospective study revealing that a bacteriological cure was not achieved in up to 17% of cases (Priest and Peacock, 2005). Possible explanations for this persistence include the increasing incidence of antibiotic resistance in *S. aureus* and *Salmonella* spp. (Workman et al., 1996; Heseltine, 2000), with the appearance of isolates that are even resistant (Boneca and Chiosis, 2003) or less susceptible (Tenover et al., 2001) to vancomycin, and the formation of staphylococcal bacterial biofilms that may render these organisms less susceptible to host immune attack (as reviewed in Brady et al., 2008; Hanke and Kielian, 2012). However, recent *in vitro* studies have demonstrated that neutrophils can recognize biofilms and respond by phagocytosis and release of lactoferrin, elastase, and DNA suggesting that staphylococcal biofilms are not inherently resistant to neutrophil-mediated destruction, although it remains to be determined how effectively these cells attack biofilms *in vivo* (Meyle et al., 2010). Furthermore, these explanations fail to fully account for the observation that up to 50% of “cured” osteomyelitis cases display infection-related sequelae. Indeed, patients can have recurrent attacks of osteomyelitis following the completion of treatment regimens even when causative organisms can no longer be isolated from infection sites (Priest and Peacock, 2005).

An alternative explanation for these phenomena may lie in the ability of the causative agents of osteomyelitis to invade and reside within resident bone cells. An intracellular lifestyle provides advantages to a variety of microbes that include access to host cell metabolites (as discussed in Ray et al., 2009; Wright and Nair, 2010; Friedrich et al., 2012). Importantly, internalization also provides a means of protection against neutrophil and antibody-mediated immune responses, and mitigates therapeutic interventions by limiting exposure to antibiotics. *Salmonella* species are intracellular bacterial pathogens and this organism is recognized to invade epithelial cells (Rosenshine et al., 1994; Jones and Falkow, 1996; Ohl and Miller, 2001). Importantly, we have demonstrated that this bacterium can invade primary osteoblasts (Bost et al., 2000). In contrast, staphylococci have traditionally been regarded as non-invasive pathogens that damage host bone cells after adhering to the extracellular matrix (Nair et al., 1996). However, it is becoming increasingly apparent that these organisms can be internalized by cultured osteoblasts and can persist intracellularly (Hudson et al., 1995; Ellington et al., 1999, 2003, 2006; Jevon et al., 1999; Ahmed et al., 2001). Osteoblasts appear to be active in this invasion process as evidenced by the requirement that these cells, but not bacteria, be alive for internalization to occur (Hudson et al., 1995; Ellington et al., 1999). Furthermore, reorganization of the osteoblast cytoskeleton and receptor-mediated endocytosis are utilized in the internalization process (Ellington et al., 1999; Jevon et al., 1999). It is important to note that bacteria released following lysis or trypsinization of *S. aureus*-containing human osteoblasts are viable and are capable of invading other osteoblasts (Ellington et al., 2003). In addition, significant time-dependent changes in the structure of *S. aureus* can occur after as little as 12 h exposure to an intracellular environment that render the organisms less sensitive to antibiotics capable of penetrating eukaryotic cells (Ellington et al., 2006). Interestingly, the ability of bacteria to invade osteoblasts may also direct the ability of this organism to form biofilms and it has been suggested that invasion promotes a biofilm-like surface on *S. aureus* that correlates with antibiotic resistance (Brady et al., 2008). As such, these host evasion mechanisms may be intimately related (Brady et al., 2008). Such findings may explain why antibiotic treatment can reduce the number of viable bacteria in animal models of staphylococcal osteomyelitis but does not reliably sterilize infected bone (Monzón et al., 2001). Bacteria sequestered inside the osteoblast may therefore provide a reservoir of bacteria and contribute to recurrent chronic osteomyelitis that often occurs despite the presence of antibiotics and a seemingly adequate humoral response (Lew and Waldvogel, 2004).

**Figure 1:**
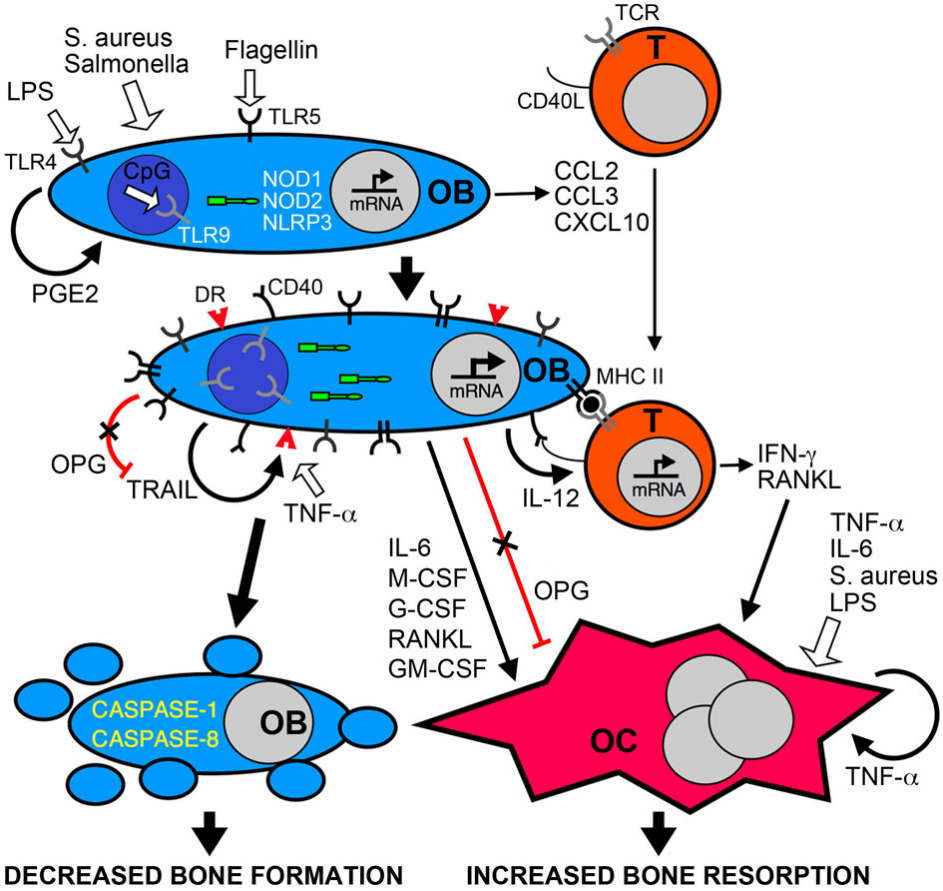
**Bacterial infection leads to inflammatory bone loss resulting from increased formation and/or activity of bone resorbing osteoclasts, and a decrease in the number of bone forming osteoblasts.** Osteoblasts (OB) can recognize bacteria and their components such as LPS, flagellin, or unmethylated CpG DNA motifs (CpG) via cell surface pattern recognition receptors including TLRs 4, 5, and 9, and the cytosolic sensors NOD1, NOD2, and NLRP3. These microbial sensors can directly or indirectly (via PGE2 autocrine/paracrine signaling) induce the rapid release of chemokines such as CCL2, CCL3, and CXCL10 to recruit leukocytes including T-lymphocytes (T) to the site of infection. Bacterially stimulated osteoblasts show elevated levels of these bacterial sensors and can also express cell surface molecules including MHC class II and CD40 that can elicit antigen-specific activation of infiltrating T-cells. Bacteria and their components can directly induce the formation and activity of osteoclasts (OC) or indirectly activate these cells via the autocrine/paracrine actions of released TNF-a. In addition, activated T-cells and osteoblasts produce an array of molecules including RANKL, IL-6, IFN-γ and colony stimulating factors (M-CSF, G-CSF, GM-CSF) that can promote osteoclastogenesis and bone resorption, and activation of osteoblasts decreases the production of the RANKL decoy receptor OPG. Finally, bacteria can induce the apoptotic death of osteoblasts either directly via the NLRP3 inflammasome, or indirectly via the autocrine/paracrine actions of released TRAIL interacting with death receptors (DR) expressed on the surface of challenged cells. Since OPG also functions as a decoy receptor for TRAIL, the decreased production of this molecule by infected osteoblasts could exacerbate TRAIL-induced apoptosis leading to decreased numbers of this bone forming population and greater bone loss at sites of infection.

In addition to the synthesis of new bone matrix, osteoblasts produce soluble factors that regulate the formation and activity of osteoclasts. For example, osteoblasts are an important source of receptor activator of NF-kB ligand (RANKL) and osteoprotegerin (OPG), critical regulators of osteoclast development and function (Wada et al., 2006). RANKL interacts with its receptor RANK on osteoclast progenitor cells and plays a central role in promoting osteoclast formation and activity (Hsu et al., 1999; Kong et al., 1999a,b; Kim et al., 2000; Li et al., 2000; Wada et al., 2006). In contrast, OPG functions as a decoy receptor for RANKL and limits osteoclastogenesis (Wada et al., 2006). Interestingly, causative agents of periodontal infections such as *Porphyromonas gingivalis* have been found to induce the RANKL expression in osteoblasts (Okahashi et al., 2004) and elevated levels of this molecule have been found in osteomyelitis-associated bone lesions (Montonen et al., 2006). While such RANKL production may be attributable to the activity of resident bone cells, it should be noted that activated T-lymphocytes also produce this cytokine (Anderson et al., 1997; Horwood et al., 1999; Hsu et al., 1999). RANKL production by osteoblasts and T-cells can be stimulated by the inflammatory cytokines interleukin (IL)-1β and tumor necrosis factor-α TNF-α (Walsh and Choi, 2003) that are detectable in infected bone tissues of osteomyelitis patients (O'Keefe et al., 1997). In addition, both of these inflammatory mediators have been demonstrated to concomitantly depress OPG expression in osteoblasts and we have shown that *S. aureus* challenge similarly inhibits osteoblast production of this RANKL decoy receptor (Young et al., 2011). This then provides another mechanism that can favor bone resorption at sites of infection. Finally, Choi et al. (2005) have shown that cyclooxygenase-2 (COX2) inhibitors can inhibit *P. gingivalis*-induced RANKL expression by osteoblasts and restore OPG levels. This observation is in agreement with our own recent studies demonstrating that *S. aureus* elevates RANKL production by osteoblasts and this effect is similarly abolished by a specific COX2 inhibitor (Somayaji et al., 2008). These findings suggest that osteoblasts produce PGE2 that acts in a paracrine or autocrine manner to induce RANKL release and limit OPG production, creating a microenvironment that favors bone resorption (Choi et al., 2005). Together, these data suggest that bacterially challenged osteoblasts can promote the formation and activity of osteoclasts leading to a net loss of bone at sites of infection (as summarized in Figure 1).

But the activities of osteoblasts may not be limited to the synthesis of bone matrix components and the regulation of osteoclast activity. There is increasing evidence that osteoblasts may directly contribute to inflammation in infected bone tissue. Studies from our group and others have demonstrated that isolated osteoblasts utilize members of the toll-like receptor (TLR) and nucleotide-binding oligomerization domain-containing (NOD)-like receptor (NLR) families of innate immune receptors to detect the presence of microbial products (Gasper et al., 2002; Madrazo et al., 2003; Marriott et al., 2005; Chauhan and Marriott, 2010) (Figure 1). The activation of these sensors precipitates transcription factor activation and leads to the release of inflammatory cytokines and the expression of cell surface antigen presenting and co-stimulatory molecules (Barton and Medzhitov, 2003). Consistent with this observation, our *in vitro* studies show that *S. aureus* is a potent stimulus for the production of soluble and cell surface molecules by isolated osteoblasts that could play key roles in the initiation and/or progression of inflammatory immune responses (Marriott, 2004). Such molecules include CXCL10 (Gasper et al., 2002) and CCL2 (Bost et al., 2001) that are potent chemoattractants for T-cells and macrophages, leukocytes frequently observed in bone tissue following infection (Stashenko et al., 1992; Bremell et al., 1994), and IL-6 (Bost et al., 1999) an often inflammatory cytokine that can directly or indirectly modulate the activity of bone-resorptive osteoclasts (Lowik et al., 1989; Ishimi et al., 1990; de la Mata et al., 1995; Greenfield et al., 1995; Hofbauer and Heufelder, 1996). Furthermore, our laboratory has demonstrated that primary osteoblasts can produce growth factors such as granulocyte macrophage colony stimulating factor (GM-CSF), macrophage colony stimulating factor (M-CSF) and granulocyte colony stimulating factor (G-CSF) following *S. aureus* exposure (Bost et al., 2000) that could increase osteoclastogenesis and promote bone resorption (Lorenzo et al., 1987; Kodama et al., 1991; Takahashi et al., 1991; Liggett et al., 1993; Povolny and Lee, 1993), and augment immune responses to this bacterial pathogen (Frenck et al., 1990; Freund and Kleine, 1992; Dale, 1995). As such, the production of these mediators by bacterially challenged osteoblasts may significantly contribute to involucrum and sequestrum formation during osteomyelitis, and exacerbate damaging inflammation.

## Modulation of leukocyte apoptosis in osteomyelitis

The interaction between *Salmonella* species and host cell types often results in the death of mammalian cells but these bacteria appear to do so by host cell-type specific mechanisms (as reviewed in Fink and Cookson, 2007) and the mechanisms underlying host cell death by such Gram-positive bacterial pathogens have been extensively reviewed elsewhere (Ulett and Adderson, 2006). While *Salmonella* induces apoptosis in epithelial cells, invasion of macrophages rapidly triggers caspase-1-dependent programmed cell death, or pyroptosis, in a salmonella pathogenicity island-1 type III secretion system and flagella dependent manner (Fink and Cookson, 2007). Since caspase-1-deficient mice are more susceptible to salmonellosis, such pyroptotic death has generally been considered to be a protective response to infection (Fink and Cookson, 2007). In contrast, *Brucella abortus*, an intracellular zoonotic pathogen that causes osteomyelitis in humans, elicits host macrophage apoptosis following invasion (Cha et al., 2013). However, apoptosis only occurs after a period of bacterial replication and so such cell death appears to favor the pathogen by eliminating immune cells. Furthermore, it should be noted that some Gram-positive pathogens of bone including streptococci also show a capacity to escape in a viable form from macrophages in an NO-dependent manner (Ulett and Adderson, 2005).

While several causative agents of osteomyelitis can induce programmed cell death of infiltrating macrophages, delayed neutrophil apoptosis is a characteristic feature of human osteomyelitis arising from either Gram-negative or Gram-positive organisms (as discussed in Ocaña et al., 2008). Neutrophils from osteomyelitis patients exhibit less spontaneous apoptosis than that seen in cells from healthy donors (Asensi et al., 2004). Interestingly, the serum of osteomyelitis patients has been found to significantly reduce apoptosis rates over a 12 h time period in isolated neutrophils (Asensi et al., 2004). This effect has been attributed to the elevated IL-6 levels seen in patient's sera as these anti-apoptotic effects could be reversed with IL-6 neutralizing antibodies or mimicked with exogenous IL-6 (Asensi et al., 2004). Such cytokine-induced neutrophil apoptosis inhibition has been demonstrated following infection with the Gram-positive organism, *S. aureus*, or the Gram-negative bacterium, *Escherichia coli*, and has also been observed following challenge with lipoteichoic acid (LTA) or LPS, bacterial ligands for TLR2 and TLR4 pattern recognition receptors, respectively (Ocaña et al., 2008). This auto-induced reduction in neutrophil apoptosis correlates with an altered ratio of pro-apoptotic B-cell lymphoma (Bcl)2–associated X protein (Bax) to anti-apoptotic Bcl-extra large (Bcl-xL) expression in these cells, and this is notable since the loss-of-function Bax promoter polymorphism A allele has been found to be more frequent in osteomyelitis patients (Ocaña et al., 2008). The neutrophils in such patients express less Bax and, accordingly, lower rates of apoptosis. Similarly, polymorphisms in the gene encoding TLR4, but not TLR2, have been identified as risk factors for chronic Gram-negative bacterial osteomyelitis (Montes et al., 2006). Neutrophils isolated from osteomyelitis patients with TLR4 polymorphisms show less LPS-induced activation of the key pro-inflammatory transcription factor NF-kB, reduced IL-6 production, and a lower induced reduction in apoptosis (Montes et al., 2006). It is conceivable that inflammatory cytokine-induced lengthening of neutrophil lifespan represents an attempt by the host to augment bacterial killing at the site of infection. However, limiting leukocyte apoptosis in this manner may ultimately be detrimental to the host due to the exacerbation of inflammatory damage (Ocaña et al., 2007).

## Modulation of osteoclast formation and survival in osteomyelitis

While bacteria or their products can directly destroy bone tissue, they can also indirectly elicit bone loss by increasing the formation and function of bone-resorbing osteoclasts (Chung et al., 2006; Maruyama et al., 2006). Indeed, osteoclast responses in *S. aureus*-infected tissue differ markedly from those seen in sterile bone trauma in that these cells behave as acute inflammatory responders with substantial activity at the margins of the infected site and adjacent uninjured tissue (Pesanti and Lorenzo, 1998). Osteoclastogenesis and osteoclast activity can be up-regulated by soluble mediators produced by infiltrating leukocytes such as macrophages and neutrophils. For example, the capsular-like polysaccharide antigen of *A. actinomycetemcomitans*, an organism implicated in juvenile periodontitis, stimulates the production of IL-1α that, in turn, promotes osteoclast formation and bone resorption (discussed in Yamamoto et al., 1999). Similarly, surface-associated material extracted from *S. aureus* has been shown to stimulate osteoclastogenesis and pit formation on dentine slices in a TNF-α and IL-6 dependent manner (Meghji et al., 1998). Such induction by cytokines generally considered to be inflammatory could, in part, explain the large numbers of osteoclasts that are typically associated with infarcted bone in osteomyelitis.

However, the formation and bone resorbing functions of osteoclasts are regulated in large part by osteoblasts and a substantial body of evidence has accumulated to show that bacterially challenged osteoblasts produce an array of soluble mediators that can promote osteoclastogenesis and osteoclast activity (as summarized in Figure 1). Recently, Gram-negative bacterial LPS has been demonstrated to induce osteoclastogenesis and reduce bone volume following in vivo administration, and this effect was blocked by co-administration of OPG (Ochi et al., 2010). While most cultured mononuclear osteoclasts die within 24 h in the absence of stimuli, both RANKL and bacterial LPS support survival and induce differentiation into multinuclear cells, providing a potential mechanism underlying LPS-induced bone loss (Suda et al., 2002). Such an effect does not appear to be limited to Gram-negative bacterial products, as *S. aureus* binding to osteoblasts can induce the release of soluble RANKL by these cells (Widaa et al., 2012). Furthermore, biofilm components from a clinical *S. aureus* strain have recently been demonstrated to induce osteoblast RANKL production and increase the RANKL/OPG ratio in culture media (Sanchez et al., 2013). In particular, staphylococcus protein A (SpA) has been reported to bind to TNF receptor 1 (TNFR1) on osteoblasts (Claro et al., 2013), and *S. aureus*-induced increases in osteoblast RANKL expression are not seen with SpA mutants (Claro et al., 2011). However, one study suggests that *S. aureus* surface-associated material can promote osteoclast formation *in vitro* in a RANKL independent manner (Lau et al., 2006), while another indicates that LPS-induced bone resorption can occur independent of RANKL or the inflammatory mediators IL-1β or TNF-α (Suda et al., 2002). As such, it is possible that bacterial products can have both direct and indirect effects on osteoclast activity in inflammatory bone loss.

It is important to note that some bacterial pathogens appear to manipulate bone homeostasis in a species-specific manner. For example, *P. gingivalis* culture products including hemoglobin receptor protein can suppress RANKL-induced *in vitro* osteoclastogenesis (Fujimura et al., 2006). It seems likely that differences in the manipulation of bone tissue reflect distinct bacterial survival and/or dissemination strategies. While net resorption of bone may favor *S. aureus* and *Salmonella* infections, rapid and extensive destruction of alveolar bone would be anticipated to result in tooth loss and the elimination of the gingival crevice that is an anatomical niche for periodontal pathogens such as *P. gingivalis*. A suppressive effect of this organism on osteoclastogenesis may, therefore, permit chronic infection (Fujimura et al., 2006).

## Induction of apoptosis in bone forming osteoblasts during osteomyelitis

In addition to the increased formation and activity of bone resorbing osteoclasts in osteomyelitis, inflammatory bone loss may also result from the elimination of the cells responsible for new bone matrix deposition following infection. In support of this notion, reduced expression of markers associated with differentiated osteoblasts including alkaline phosphatase activity, collagen type I production, and mineralized nodules, is seen in alveolar bone tissue from patients with periodontal disease (Mori et al., 2007). Importantly, work from our laboratory and our collaborators have demonstrated that bone-forming osteoblasts undergo apoptotic cell death following infection with the principle causative agents of osteomyelitis, *S. aureus* and *Salmonella* (Tucker et al., 2000; Alexander et al., 2003). We have shown that *Salmonella enterica* can elicit significant apoptotic cell death as rapidly at 24 h post-infection. Interestingly, we have found that intracellular invasion is required for maximal induction of apoptosis in osteoblasts as evidenced by the sensitivity of this response to a pharmacological inhibitor of endocytosis and a significantly smaller apoptotic response in this cell type to invasion deficient *Salmonella* strains (Marriott et al., 2005; McCall et al., 2008). As such, inflammatory bone loss during osteomyelitis may result from the elimination of the cells responsible for new bone matrix deposition in addition to the increased numbers and activity of bone resorbing osteoclasts at sites of bacterial infection (Nair et al., 1996). Along these lines, it is becoming apparent that the causative agents of osteomyelitis are capable of inducing continuous apoptosis of bone lining osteoblasts by activating intrinsic and extrinsic cell death pathways to further uncouple bone formation and resorption.

## Direct induction of osteoblast cell death following bacterial challenge

Clinical *S. aureus* strain biofilm components have been shown to inhibit osteoblast differentiation and reduce viability with increased rates of apoptosis (Sanchez et al., 2013). Similarly, *S. aureus* binding to osteoblasts inhibits de novo bone formation by preventing expression of key products including alkaline phosphatase, collagen type I, osteocalcin, and osteopontin (Widaa et al., 2012). SpA appears to underlie this effect as this product can directly bind osteoblasts, inhibit proliferation and mineralization, and initiate apoptosis in this cell type (Claro et al., 2011). A role for SpA binding to osteoblasts in inducing apoptosis is supported by the observation that these effects are not seen with SpA deficient bacterial mutants (Claro et al., 2011; Widaa et al., 2012). Similarly, *A. actinomycetemcomitans* extracts can also induce the death of osteoblast-like cells (Morimoto et al., 1999). Again, a bacterial protein product appears to underlie this phenomenon as cycloheximide-treated *A. actinomycetemcomitans* fails to induce osteoblast death, and heat or trypsin treatment of bacterial extracts neutralizes this effect (Morimoto et al., 1999). Although, it should be noted that at least one study suggests that *A. actinomycetemcomitans*-induced osteoblast apoptosis can be mediated by a capsular-like polysaccharide (Yamamoto et al., 1999). Interestingly, while *P. gingivalis* lipids can inhibit osteoblast differentiation and activity, they do not significantly alter osteoblast viability (Wang et al., 2010), but binding between the fimbriae of this organism and the host cell integrin alpha5beta1 can initiate apoptosis in this cell type (Zhang et al., 2013). However, such induction appears to be a delayed effect as *P. gingivalis* prolongs cell life prior to host cell destruction (Zhang et al., 2013), presumably to permit bacterial replication within the osteoblast.

We have demonstrated that osteoblasts express members of the TLR family of pattern recognition receptors and that these cell surface sensors can detect the presence of extracellular bacteria and their products (Kikuchi et al., 2001; Gasper et al., 2002; Madrazo et al., 2003). However, our studies indicate that bacterial components, UV-inactivated bacteria and invasion defective strains of bacteria are far less effective in eliciting the osteoblast immune functions than viable wild type bacteria (Bost et al., 1999, 2000, 2001). Furthermore, Thammasitboon et al. (2006) have demonstrated that bacterial components such as LPS fail to elicit apoptosis in osteoblasts. Consistent with these findings, we have shown that cytochalasin significantly attenuates inflammatory cytokine production by invasive *Salmonella* (McCall et al., 2008). These observations indicate that invasion is required for optimal osteoblast responses and suggest that the cell surface TLRs are not the only means by which these cells perceive bacterial pathogens. This phenomenon might be explained by our finding that osteoblasts express NOD1 and NOD2 (Marriott et al., 2005), two members of the NLR family of receptor proteins that can serve as intracellular sensors for bacterial peptidoglycans and initiate pro-inflammatory mediator production (as reviewed in Ting and Davis, 2005; Strober et al., 2006).

The importance of bacterial invasion in the initiation of osteoblast apoptosis is underscored by the demonstration that the presence of intracellular *S. aureus* causes rounding of this cell type and chromatin condensation, DNA laddering, and DNA strand breaks in the nuclei (Tucker et al., 2000). Furthermore, *S. aureus-*induced apoptosis in human osteoblasts is markedly attenuated following inhibition of cellular invasion (Ning et al., 2011). Similarly, the Gram-negative bacteria *P. gingivalis* and *B. abortus*, causative agents of periodontitis and osteomyelitis, respectively, are capable of invading osteoblasts, although these organisms appear to elicit delayed apoptosis thereby permitting intracellular replication (Wang et al., 2010; Scian et al., 2012; Baldi and Giambartolomei, 2013; Zhang et al., 2013). Finally, similar to inflammatory mediator production, our studies indicate that invasive bacteria are more effective at inducing osteoblast apoptosis with *S. aureus* or *S. typhimurium* SB300 being significantly more potent stimuli for murine osteoblast cell death than the invasion defective species, *S. carnosus* and *S. typhimurium* SB136 (Marriott et al., 2005).

While NOD1 and NOD2 can initiate, augment, or reduce inflammatory mediator production by a variety of cell types (as reviewed in Inohara and Nunez, 2003), these cytosolic proteins are not widely recognized to participate in the induction of apoptosis. In contrast, two related receptors, NLR family CARD domain containing 4 (NLRC4) and NLR family pyrin domain containing 3 (NLRP3), have been implicated in the induction of cell death in response to bacteria and/or their components (Gumucio et al., 2002; Mariathasan et al., 2004, 2006; Sutterwala et al., 2006). Both of these molecules can associate with an adaptor protein, apoptosis-associated speck-like protein (ASC) to elicit caspase-1 and caspase-8 activation (Hasegawa et al., 2005; Mariathasan, 2007), enzymes that demonstrate elevated activity in osteoblasts following bacterial challenge (Marriott et al., 2002; Alexander et al., 2003). We have recently investigated the functional expression of NLRP3 and NLRC4 in resting and *Salmonella* exposed cultures of primary murine and human osteoblasts in an attempt to identify the mechanisms linking intracellular invasion to bacterially-induced cell death. We demonstrated that osteoblasts constitutively express NLRP3, but not NLRC4, and such expression was modestly increased following infection with wild type *S. enterica* (McCall et al., 2008). This finding was in contrast with the significant decrease in NLRP3 protein levels seen in osteoblasts following exposure to an invasion defective strain. In addition to showing that osteoblasts possess robust constitutive levels of NLRP3, we demonstrated that these cells also express ASC. While constitutive levels of ASC were low in resting osteoblasts, a marked elevation in expression was observed in cells following *Salmonella* infection (McCall et al., 2008).

Taken together, the conserved expression of NLRP3 and ASC in both mouse and human osteoblasts, and the sensitivity of such expression to bacterial challenge, provides circumstantial evidence of a role for these cytosolic proteins in osteoblast responses to intracellular pathogens. However, we have more directly verified NLRP3 functionality in bacterially challenged osteoblasts (McCall et al., 2008). First, we have shown that NLRP3 associates with ASC following exposure to *Salmonella* as determined by co-immunoprecipitation. Second, we have demonstrated that NLRP3 expression knockdown by siRNA attenuates *Salmonella*-induced changes in transcription factor activity. Third, we have found that osteoblasts derived from NLRP3 deficient animals produce less IL-6 than cells derived from wild type mice. As such, these findings confirm that NLRP3 mediates, at least in part, osteoblast responses to this intracellular bacterial pathogen.

Given that osteoblasts constitutively express NLRP3 and ASC, and the finding that NLRP3 can associate with ASC to elicit caspase-1 (Gumucio et al., 2002; Dowds et al., 2004; Kanneganti et al., 2006; Mariathasan et al., 2006) and perhaps caspase-8 activation (Hasegawa et al., 2005), enzymes that demonstrate elevated activity in osteoblasts following bacterial challenge (Marriott et al., 2002; Alexander et al., 2003), this cytosolic NLR may represent an important mechanism underlying osteoblast apoptosis following exposure to intracellular bacterial pathogens. This hypothesis is supported by our observation that *Salmonella*-induced decreases in NF-kB activity are markedly attenuated in osteoblasts following siRNA-induced NLRP3 knockdown (McCall et al., 2008), since this transcription factor has been reported to mediate anti-apoptotic effects in a variety of cell types (You et al., 2001; Lu et al., 2004; Munshi et al., 2004). Furthermore, we have shown that *Salmonella*-induced caspase-1 activation is absent in osteoblasts derived from NLRP3 deficient animals (McCall et al., 2008). More importantly, we have demonstrated that such cells exhibit significantly less apoptotic cell death following *Salmonella* infection than osteoblasts derived from wild type animals (McCall et al., 2008). These findings differ from studies in macrophages (Mariathasan et al., 2004, 2006) where NLRP3 is not required for *Salmonella*-induced caspase-1 activation, IL-1β production, or cell death. Instead, these functions are predominantly mediated by NLRC4 (Mariathasan et al., 2004, 2006). In osteoblasts, NLRP3 is required for caspase-1 activation and for a portion of *Salmonella*-induced cell death, while NLRC4 is not detectable in these cells. Furthermore, earlier work from our laboratory has indicated that neither the precursor nor the mature form of IL-1β is produced by infected osteoblasts (Marriott et al., 2002). Together these studies underscore the differences between osteoblasts and macrophages and show that at least one of these is a differential reliance on NLRP3 vs. NLRC4 in cell death following *Salmonella* infection. This illustrates an additional level of complexity in the control of cellular functions by NLR family members.

While further experimentation is required to definitively conclude that ASC and alterations in NF-kB activity are essential mechanistic elements in NLRP3-mediated apoptosis in bacterially challenged osteoblasts, these studies indicate that this NLR represents an important component underlying the direct initiation of apoptosis in this bone-forming cell type following challenge with intracellular bacterial pathogens and could, therefore, be a major contributory factor to bone loss at sites of infection.

## Indirect induction of osteoblast cell death via leukocyte/bone cell derived mediators

The extrinsic pathway of programmed cell death begins with ligation of a particular group of cell surface receptors and leads to recruitment of adaptor molecules and the activation of a caspase cascade that results in cellular disassembly. This apoptotic pathway is critically regulated by members of the TNF family of cytokines and receptors in many cell types (Cosman, 1994; Lynch et al., 1994; Smith et al., 1994). Ligands such as TNF-α and Fas ligand have the ability to interact with death domain containing receptors to initiate apoptosis and conditioned medium from LPS-treated macrophage-like cells has been shown to induce osteoblast apoptosis in a TNF-α dependent manner (Thammasitboon et al., 2006). Similarly, the supernatants from *B. abortus*-infected macrophages can induce osteoblast apoptosis in a TNF-α-dependent manner (Scian et al., 2012), which could therefore contribute to the bone and joint destruction seen in patients with osteoarticular complications of brucellosis. However, it is important to note that the events that occur in vivo are likely to be more complex as illustrated by the observation that LPS-induced TNF-α production only provokes in situ osteoblast apoptosis when the TNFR1 signal is negated, suggesting that this receptor can also regulate osteoblast survival (Ochi et al., 2010).

TNF-related apoptosis inducing ligand (TRAIL) is also a member of the TNF superfamily and this soluble protein has the unique ability to interact with five different receptors. TRAIL can interact with death receptor (DR)4 (also known as TRAIL R1) (Pan et al., 1997), DR5 (also known as TRAIL R2 and TRAIL receptor inducer of cell killing-2) (Schneider et al., 1997), DcR1 (also known as TRAIL R3 and TRAIL receptor without an intracellular domain) (Degli-Esposti et al., 1997), DcR2 (also known as TRAIL R4 and TRAIL receptor with a truncated death-domain) (Pan et al., 1998), and finally soluble OPG (Emery et al., 1998). Of these receptors, only DR4 and DR5 in humans and DR5 in mice have functional cytoplasmic death-domains and are capable of inducing apoptosis, while DcR1 and DcR2 appear to function as decoy receptors (Clancy et al., 2005). Death domain-containing receptors trimerize to allow TRAIL to bind and trigger caspase 8 activation that directly activates caspases 3 and 7 via the extrinsic pathway (Clancy et al., 2005). Although it should be noted that that TRAIL has been also been suggested to activate the intrinsic pathway through the activation of BH3 interaction-domain death agonist (Bid) (Suliman et al., 2001; Sinicrope and Penington, 2005). Both of these pathways lead to caspase 3 activation and cell death (Suliman et al., 2001). In contrast, OPG appears to function as a soluble decoy receptor for both TRAIL and the potent osteoclastogenic factor, RANKL. As such, excessive TRAIL secretion in bone tissue would be anticipated to decrease the amount of OPG available to bind RANKL, leading to increased osteoclastogenesis and tissue loss (as illustrated in Figure 1).

In a recent study (Young et al., 2011), we have demonstrated that murine osteoblasts constitutively express mRNA encoding the sole murine death-domain containing TRAIL receptor, DR5, and we have shown that resting cultures of these cells contain robust levels of the DR5 protein. These observations are consistent with previous reports that human osteoblast-like cells express mRNA encoding DR4 and DR5 (Atkins et al., 2002). However, we have shown that DR5 expression on the surface of murine osteoblasts is restricted to cells exposed to either *S. aureus* or *Salmonella* (Young et al., 2011). Similarly, we confirmed that both of the death-domain containing TRAIL receptors on human cells are only present on primary human osteoblasts following bacterial challenge and the conserved nature of this response supports the biological significance of such expression on infected cells (Young et al., 2011). In the same work, we showed that the robust constitutive production of the decoy TRAIL receptor OPG by resting osteoblasts was markedly attenuated following infection with either *S. aureus* or *Salmonella* (Young et al., 2011). While other studies indicate that serum OPG levels increase following systemic bacterial LPS administration (Maruyama et al., 2006), our results are consistent with reports that the causative agents of periodontitis and LPS can similarly decrease OPG expression by murine osteoblasts (Chung et al., 2006; Mori et al., 2007). Furthermore, it should be noted that bone tissue is relatively poorly vascularized and bone infections are more commonly associated with Gram-positive bacterial species such as *S. aureus*, infections that do not result in the development of endotoxic shock. As such, changes in OPG expression by resident bone cells are likely to be more physiologically relevant in local bone infections than the systemic effects elicited by bacterial endotoxins. As such, these data suggest that the principle causative agents of osteomyelitis can elicit an imbalance in the expression of death-inducing and decoy TRAIL receptors by osteoblasts, with the induced expression of DR5 and/or DR4 on the surface of infected cells and a concomitant decrease in the production of the decoy receptor, OPG.

Osteoblasts isolated from alveolar bone of patients with periodontal disease have been demonstrated to have higher sensitivity to the apoptosis-inducing actions of TRAIL than cells isolated from healthy donors (Mori et al., 2009). In our recent work, we tested the sensitivity of primary osteoblasts to exogenously administered recombinant TRAIL at rest or following challenge with either *S. aureus* or *Salmonella* (Young et al., 2011). Consistent with the absence of death-inducing TRAIL receptors on uninfected osteoblasts and previous reports with unstimulated human osteoblast-like cells (Atkins et al., 2002; Bu et al., 2003), we showed that TRAIL fails to activate apoptosis signaling pathways in uninfected primary osteoblasts (Young et al., 2011). However, acute bacterial exposure sensitizes osteoblasts to TRAIL-mediated cell death as shown by the ability of exogenous TRAIL to augment apoptotic volume decreases and caspase-8 activation following *S. aureus* or *Salmonella* infection (Alexander et al., 2003; Young et al., 2011). While increased TRAIL-induced apoptosis is only correlative with the observed induction of cell surface death receptor expression, we have previously documented that TRAIL neutralizing antibodies can significantly inhibit bacterially-induced osteoblast apoptosis as assessed by caspase-8 activation and annexin V staining (Alexander et al., 2003). Together, these results support the contention that the induced expression of death receptors renders these cells sensitive to the apoptotic actions of TRAIL, and provide a potential mechanism underlying the reported increase in TRAIL sensitivity of human alveolar osteoblasts isolated from patients with periodontitis (Mori et al., 2009) and transformed osteoblasts (Atkins et al., 2002).

Several studies have shown that human osteoblast-like cells express TRAIL (Atkins et al., 2002; Bu et al., 2003) and work from our laboratory has demonstrated that exposure of mouse and human primary osteoblasts to *S. aureus* or *Salmonella* induces TRAIL expression by these cells (Alexander et al., 2001). Furthermore, we have also shown that bacterial infection enhances RANKL expression and release by osteoblasts (Somayaji et al., 2008). Since RANKL binds OPG with a similar binding affinity to that of TRAIL (23 and 45 nM for RANKL and TRAIL, respectively (Vitovski et al., 2007), this molecule could further reduce levels of OPG available to bind and neutralize TRAIL. Accordingly, a scenario could be envisaged in which bacterially challenged osteoblasts express TRAIL and RANKL while concomitantly decreasing the production of the decoy receptor OPG and upregulating cell surface death receptor expression. The increased production of this death receptor ligand, in concert with reduced OPG production and bioavailability, would facilitate TRAIL activity and result in the death of infected osteoblasts, providing an additional mechanism whereby bacterial pathogens elicit bone destruction during diseases such as osteomyelitis (as shown in Figure 1).

## Concluding remarks

The inflammatory bone loss associated with osteomyelitis can result from the direct destruction of bone tissue by bacteria and their products. Furthermore, significant bone loss is likely to result from the recruitment and activation of infiltrating leukocytes such as neutrophils and macrophages. The direct activation of these cells by bacteria via an array of cell surface and cytosolic pattern recognition receptors, and the presence of an inflammatory milieu at the site of infection, results in the production of degradative enzymes and cytotoxic molecules. Consistent with host-pathogen interactions at other anatomical sites, the microbes responsible for bone infections can manipulate leukocyte survival pathways in a bacterium specific manner with some organisms inducing apoptosis and/or pyroptotic cell death, while other organisms appear to promote the survival of innate immune cells, particularly neutrophils. While lengthening the life span of these cells might represent an attempt by the host to clear the infection, the unintended consequence of such a vigorous response can be extensive inflammatory bone destruction.

However, bone loss at sites of infection is primarily due to an uncoupling of the control of bone forming osteoblasts and bone resorbing osteoclasts. Bacteria or their products can directly increase the osteoclast formation and activity. In addition, inflammatory mediators produced at the site by infiltrating leukocytes or bacterially challenged osteoclasts themselves can further promote osteoclastogenesis and bone resorption. Importantly, the production and activity of osteoclasts is critically regulated by osteoblasts, and these cells have recently been found to respond to the principle causative agents of osteomyelitis via TLR and NLR family members. Osteoblasts exposed to bacteria express an array of soluble and cell surface molecules that have the potential to promote immune responses and directly or indirectly enhance osteoclast activity at sites of infection.

In addition to the increased number and activity of bone resorbing osteoclasts, it is becoming apparent that bone loss during conditions such as osteomyelitis is also brought about by a decline in new bone deposition. This occurs as a result of decreased production of matrix components by osteoblasts and by increased rates of apoptosis in this cell type. While the presence of certain extracellular bacterial components may be sufficient to directly reduce osteoblast viability, there is considerable evidence that internalization of the principal causative agents of osteomyelitis is required to maximally induce apoptosis in these cells. At least a component of such cell death is attributable to osteoblast-derived TRAIL acting in an autocrine and/or paracrine manner via death domain-containing receptors that are concomitantly and exclusively expressed by infected cells. However, TRAIL-induced death is unlikely to be the sole means by which osteoblasts are eliminated in infected tissue. Indeed, rapid activation of apoptotic pathways is observed in microbe challenged osteoblasts prior to TRAIL production, and at least a proportion of this cell death is unaffected by anti-TRAIL antibodies (Alexander et al., 2001, 2003). Such cell death may result from the direct activation of cytosolic NLRP3 that is constitutively expressed by osteoblasts (McCall et al., 2008), which can activate caspase cascades and initiate apoptosis. As such, NLRP3 may act in concert with TRAIL-mediated pathways to eliminate osteoblasts in infected bone tissue.

It might be anticipated that new therapies that target the perception and initiation of inflammatory immune responses and bone-forming cell death via pattern recognition receptors would be of considerable benefit in reducing the inflammatory damage and cell death associated with bone infections. However, it is important to note that many of these observations have been made in reductive *in vitro* systems and have yet to be confirmed in vivo. Also, it is clear that important issues regarding bacteria-induced inflammatory bone loss remain unresolved. For example, it is not known whether osteocytes embedded within calcified bone can respond to bacteria, an important question given the critical role of cell type in controlling bone surface cells via the lacuna-canaliculi network (Matsuo, 2009). Furthermore, the mechanism that is the most important in the pathophysiology of bacterium-induced bone loss has not been identified, and this issue is further complicated by the disparate nature of the bacterial species that are responsible for bone diseases. As such, it is not presently known whether inhibition of osteoclast formation/activity or the prevention of osteoblast destruction would be most efficient in eliminating bacterially-induced bone loss. Finally, given that infected osteoblasts may harbor intracellular bacteria such as *S. aureus* and promote inflammatory bone damage, it is not clear whether the host would be best served by preventing osteoblast cell death or by eliminating these cells via the induction of apoptosis. Clearly, further work is warranted to resolve these issues.

### Conflict of interest statement

The author declares that the research was conducted in the absence of any commercial or financial relationships that could be construed as a potential conflict of interest.
